# Different expression patterns of VISTA concurrent with PD-1, Tim-3, and TIGIT on T cell subsets in peripheral blood and bone marrow from patients with multiple myeloma

**DOI:** 10.3389/fonc.2022.1014904

**Published:** 2022-11-10

**Authors:** Shuxin Huang, Yujie Zhao, Pengjun Liao, Jinghua Wang, Zhiyan Li, Jiaxiong Tan, Xianfeng Zha, Shaohua Chen, Yangqiu Li, Liye Zhong

**Affiliations:** ^1^ Key Laboratory for Regenerative Medicine of Ministry of Education, Institute of Hematology, School of Medicine, Jinan University, Guangzhou, China; ^2^ Department of Hematology, Guangdong Academy of Medical Sciences, Guangdong Provincial People’s Hospital, Guangzhou, China; ^3^ Department of Hematology, First Affiliated Hospital, Jinan University, Guangzhou, China; ^4^ Department of Clinical Laboratory, First Affiliated Hospital, Jinan University, Guangzhou, China

**Keywords:** MM, T cell exhaustion, VISTA, PD-1, TIM-3, TIGIT

## Abstract

V-type immunoglobulin domain-containing suppressor of T cell activation (VISTA) is considered as an immunosuppressive factor and potential therapeutic target for anticancer therapy. However, little is known about VISTA expression and its role in immunosuppression in multiple myeloma (MM). In this study, VISTA expression and co-expression with programmed cell death receptor-1 (PD-1), T cell immunoglobulin mucin-domain-containing-3 (Tim-3), and T cell immunoglobulin and ITIM domain (TIGIT) in CD3+, CD4+, CD8+, and regulatory T (Treg) cells were analyzed in patients with MM by multi-color fluorescent flow cytometry of peripheral blood (PB) and bone marrow (BM) samples from 36 patients with MM and compared to 36 PB samples and 10 BM samples from healthy individuals (HIs), which served as controls. The results demonstrated a significant increased percentage of VISTA co-expression with PD-1, Tim-3, and TIGIT in CD3+, CD4+, CD8+, and Treg cells in PB from MM patients compared with HIs. A similar trend for VISTA+CD8+ T cells was found in BM. Moreover, a trend of a high percentage on VISTA expression and co-expression in PB rather than BM was found. Furthermore, significant positive correlations existed for VISTA expression concurrent with PD-1, Tim-3, and TIGIT in T cell subsets and clinical indicators, including Revised International Staging System (R-ISS) staging of multiple myeloma, Eastern Cooperative Oncology Group (ECOG) score, and beta-2-microglobulin (β2-MG). In conclusion, higher VISTA expression concurrent with PD-1, Tim-3, and TIGIT on T cells, particularly in the PB of patients with MM, may result in T cell exhaustion and dysfunction and be closely associated with disease progression and clinical indicators. Thus, VISTA may be considered a potential target for reversing T cell exhaustion and improving T cell function in MM.

## Introduction

Multiple myeloma (MM) is the second most common hematological malignancy that is characterized by abnormal proliferation of clonal and terminally differentiated B lymphocytes in the bone marrow (BM) with clinical manifestations mainly including anemia, hypercalcemia, bone disease, and renal impairment (1). Current therapy for MM often consists of initial induction therapy, consolidation, and maintenance therapy. Despite the emergence and application of proteasome inhibitors and immunomodulators, which have made great advances in prolonging survival for MM patients in recent years, the disease remains incurable in most cases (2, 3). Additionally, monoclonal antibodies and engineered cellular therapies are rapidly making their way to the clinic, demonstrating great potential to achieve minimal residual disease negativity and restoring antitumor immunity. It is well known that T cells play a vital role in recognizing and eliminating tumor cells, and T cell dysregulation due to T cell exhaustion in MM further promotes the immune escape of malignant plasma cells. T cell exhaustion results from complex interactions of multiple immune checkpoint (IC) proteins including programmed cell death receptor-1 (PD-1), T cell immunoglobulin mucin-domain-containing-3 (Tim-3), T cell immunoglobulin and ITIM domain (TIGIT), and cytotoxic T-lymphocyte antigen-4 (CTLA-4) (4–7), and the upregulation of these IC proteins jointly contributes to T cell immunosuppression that is closely associated with disease progression and poor prognosis (8–10). Monoclonal antibodies directed against PD-1 and CTLA-4 have been demonstrated to have acceptable tolerability profiles and clinical activity in solid tumors and lymphomas. Nevertheless, clinical trial efficacies have demonstrated some limitations and relative heterogeneity in MM, which may be due to different immune microenvironments and various degrees of T cell exhaustion (11, 12). Therefore, it is worth further exploring the characteristics of T cell immunosuppression, particularly the relationship between the complex network of immunosuppressive receptors and T cell exhaustion in patients with MM.

V-type immunoglobulin domain-containing suppressor of T cell activation (VISTA), a type I transmembrane protein, is known as a unique B7 family member. It normally is expressed on myeloid hematopoietic cells and immune cells, including lymphocytes, dendritic cells, and macrophages, and participates in cell activation and functional regulation (13, 14). When VISTA is expressed on T cells, it directly inhibits the activation of CD4+, CD8+, and T cell receptor (TCR) γδ T cells and contributes to the differentiation of Tregs (15). While expressed on myeloid or antigen presenting cells, VISTA decreases the secretion of cytokines and T cell proliferation by down-regulating signal transduction pathways in T cells such as the mammalian target of rapamycin (mTOR) and mitogen-activated protein kinase (MAPK) pathways (16). Numerous studies have supported the view that the function of VISTA is not only as a receptor but also as a ligand (17, 18). In addition, abnormal VISTA expression is usually accompanied by IC proteins such as PD-1, Tim-3, and PD-L1 in tumor cells, myeloid-derived suppressor cells (MDSCs) and T cells, suggesting that tumor cells escape immune surveillance (19–21). VISTA is also involved in disease progression and can be used to predict overall survival (OS) (14, 19). For patients with melanoma, glioma, oral squamous cell carcinoma, cervical cancer, and bladder cancer, higher VISTA expression tends to be associated with poor prognosis (22–26). In contrast, several findings have shown that high levels of VISTA contribute to a favorable immune microenvironment and better overall survival for non-small-cell lung cancer (NSCLC), breast cancer, hepatocellular carcinoma, esophageal adenocarcinoma (EAC), and serous ovarian cancer (27–31). The controversial results may be due to the different expression patterns of VISTA in various tumors and involve different functions and mechanisms. As for hematologic malignancies, it has been reported that high expression of VISTA and programmed death protein ligand 1 (PD-L1) synergistically predict poor prognosis in patients with NK/T lymphoma (32). Moreover, VISTA is found to be highly expressed on MDSCs in AML, and knockdown of VISTA could reduce the MDSC-mediated inhibition of CD8+ T cell activity (21). In numerous solid tumor animal models, it has been proven that small molecule drugs or monoclonal antibodies directed against VISTA are beneficial to directly inhibiting Tregs and promoting the invasion and proliferation of tumor effector T cells as well as the secretion of cytokines, thereby enhancing the anti-tumor response (19). Furthermore, the results of a clinical trial on melanoma demonstrated higher frequencies of VISTA+ and Treg cells in patients after anti-PD-1 treatment in comparison with untreated patients, indicating that negative IC regulation by VISTA may be an important potential mechanism of acquired resistance in melanoma patients treated with anti-PD-1 (33). Similarly, VISTA offers another compensatory inhibitory pathway in prostate tumors after anti-CTLA-4 therapy (34). In summary, VISTA is considered a potential therapeutic target for anticancer therapy that plays a vital role in immunosuppression (35). However, little is known about VISTA expression and its relevant mechanisms in MM. In this study, we characterized VISTA expression and co-expression with PD-1, Tim-3, and TIGIT in T cell subsets in PB and BM from patients with MM.

## Methods

### Samples

PB and BM samples were collected from 36 newly diagnosed, untreated MM patients including 19 males and 17 females (median age: 62 years, range: 36-85 years), numbered P1 to P36. The clinical data of the patients are listed in **Supplementary Table 1**. PB samples from 36 healthy individuals (HIs), including 18 males and 18 females (median age: 58 years, range: 30-77 years), and BM samples from 10 HIs, including 5 males and 5 females (median age: 63 years, range: 20-76 years) served as controls. All samples were obtained with informed consent, and ethical approval was obtained from the Ethics Committee of School of Medicine of Jinan University and Guangdong Provincial People’s Hospital.

### Flow cytometry analysis

Extracellular staining was performed according to the manufacturer’s instructions and our previous study (36). First, cells obtained from fresh PB or BM samples were stained with surface markers including VISTA, PD-1, Tim-3, TIGIT, CD3, CD4, CD8, CD25, and CD45 and relevant isotype controls. Second, cells were fixed and permeabilized by Foxp3/Transcription Factor Staining Buffer (eBioscience, San Diego, USA) for 30 minutes at 4°C in the dark, and they were then washed twice with 1X Permeabilization Buffer. Finally, the cells were incubated with FoxP3, and relevant isotype controls for 30 minutes at 4°C in the dark. The cells were washed twice with 1X Permeabilization Buffer and resuspended in 0.5 ml staining buffer to prepare for flow cytometry analysis. A total of 30,000 CD45+CD3+T cells were acquired for analysis with a BD FACS Canto flow cytometer (BD Biosciences, San Jose, USA) and subsequent analysis by Flowjo software (Flowjo LLC, USA). Before obtaining the target CD45+CD3+ T cells, dead and sticky cells were eliminated by FSC-A/FSC-H gating. And then we analyzed the VISTA expression and co-expression with PD-1, Tim-3 and TIGIT in CD45+CD3+, CD4+CD45+CD3+, CD8+CD45+CD3+ and CD4+CD25+FoxP3+ T cells. The antibodies used for this study were purchased from BD Biosciences (San Jose, USA), BioLegend (San Diego, CA), and eBioscience (San Diego, USA). The information of antibodies is shown in **Supplementary Table 3**.

Besides, we consistently downsample CD3+ T cells number to 3000 in each sample with “downsample” plugin in Flowjo. And then, connect all downsample files in the same group (MM group or HIs group) to a whole. Finally, we used “tSNE” plugin in Flowjo software to intuitively show the difference of VISTA distribution concurrent with PD-1, Tim-3 and TIGIT cells in CD3+ T cells in MM group and HIs group.

### Statistical analysis

All data analyses were performed using SPSS software. The Mann-Whitney U test was used to analyze data between patients with MM and HIs for two independent samples, and the Wilcoxon signed-rank test was used to compare IC protein expression in T cell subsets between BM and PB for two related samples. The frequencies of the different T cell subsets are presented as medians. Correlation analyses were performed using Spearman correlation analysis. Notably, differences with a *P* < 0.05 were considered statistically significant.

## Results

### Higher frequency of VISTA co-expression with PD-1, Tim-3, and TIGIT on CD3+, CD4+, CD8+, and Treg cells in PB from MM patients

**Figure 1:**
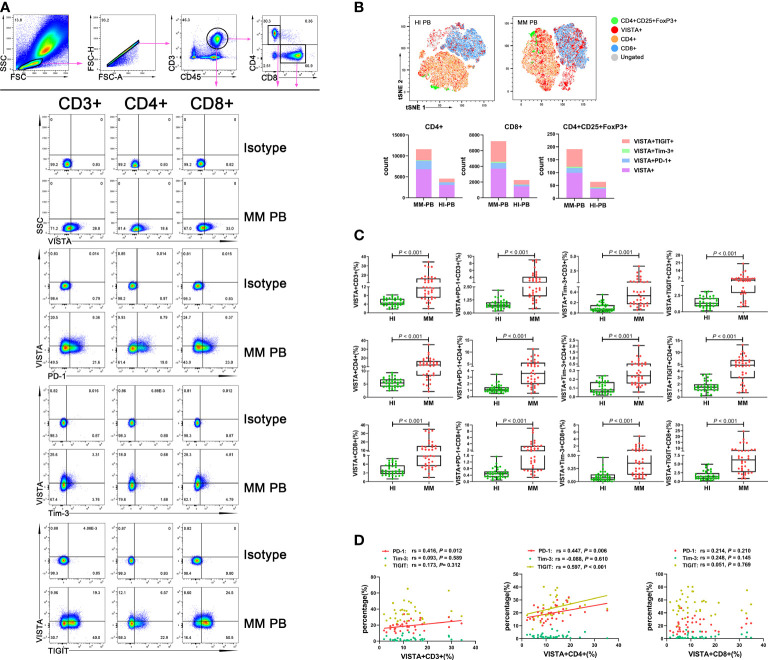
Distribution and frequency of VISTA expression and co-expression with PD-1, Tim-3, and TIGIT on T cell subsets in PB from MM patients. **(A)** Detection of VISTA, PD-1, Tim-3, and TIGIT expression on CD3+, CD4+, and CD8+ T cell subsets in a patient with MM. **(B)** tSNE clusters of the global distribution and frequency of different phenotypes of T cells in patients with MM and healthy individuals. **(C)** Comparison of the frequency of VISTA expression and co-expression with PD-1, Tim-3, and TIGIT on CD3+, CD4+, and CD8+ T cell subsets in patients with MM and healthy individuals. **(D)** Correlation between the percentages of VISTA+ and PD-1+, Tim-3+ and TIGIT+ T cells in T cell subsets in MM patients.

**Figure 2:**
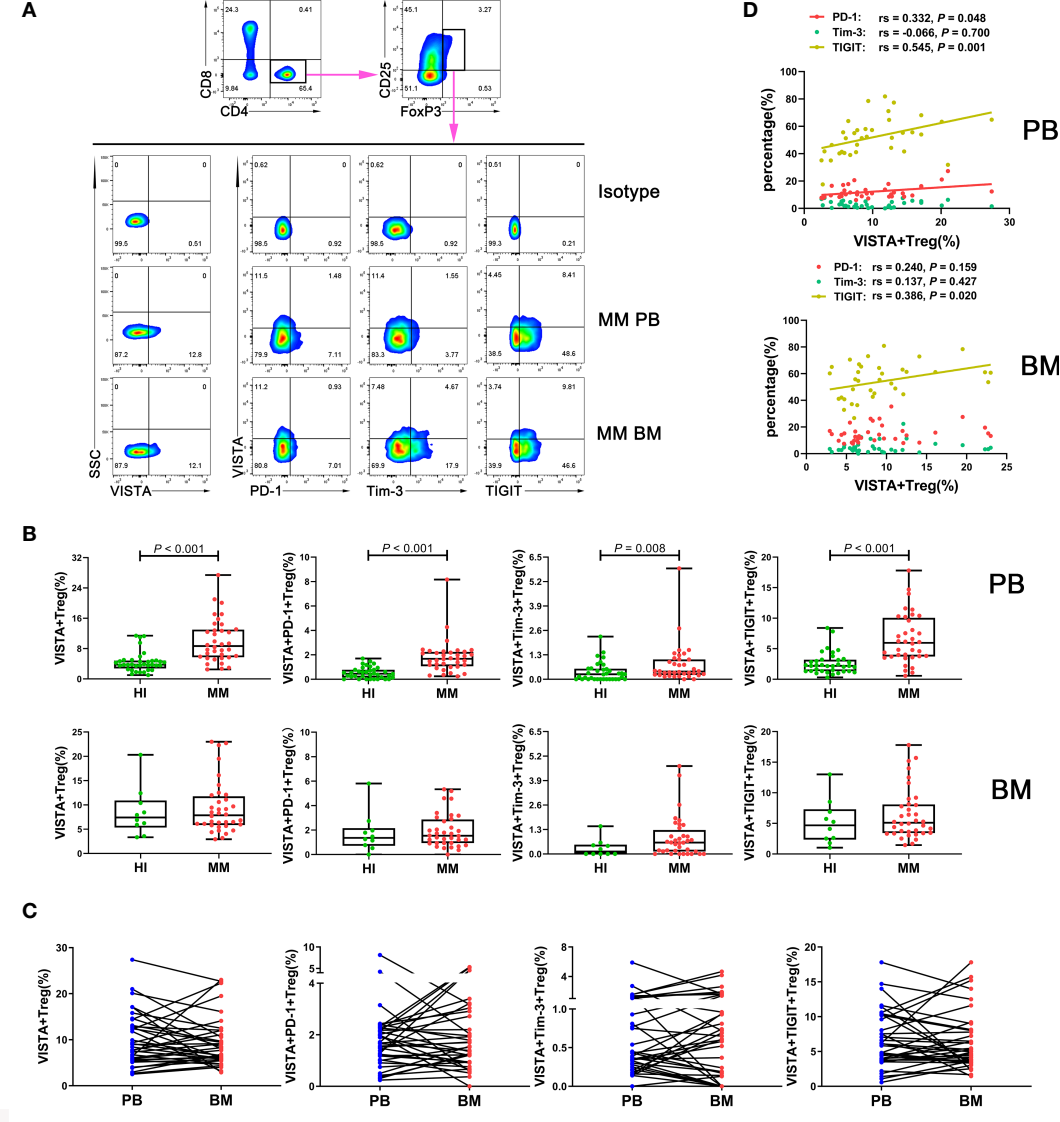
Distribution and frequency of VISTA expression and co-expression with PD-1, Tim-3, and TIGIT on Treg cells in PB and BM from MM patients. **(A)** Detection of VISTA, PD-1, Tim-3, and TIGIT expression in Treg cells in a patient with MM by flow cytometry. **(B)** Comparison of the percentage of VISTA+, VISTA+PD-1+, VISTA+Tim-3+, and VISTA+TIGIT+ Treg cells in MM and HIs. **(C)** Comparison of the percentage of the VISTA+, VISTA+PD-1+, VISTA+Tim-3+, and VISTA+TIGIT+ Treg cell subsets between PB and BM from 36 patients with MM. **(D)** Correlation between the percentages of VISTA+ and PD-1+, Tim-3+, and TIGIT+ T cells in Treg cells from MM patients.

To evaluate the VISTA+ T cell characteristics in MM, we first analyzed the percentage of VISTA on T cell subsets in PB from patients with untreated MM (**Figure 1A**). A significantly increased percentage of VISTA+CD3+ (median: 11.45 vs 4.52, *P* < 0.001), VISTA+CD4+ (median: 12.00 vs 5.45, *P* < 0.001), and VISTA+CD8+ (median: 8.79 vs 3.57, *P* < 0.001) T cells was found in MM compared with HIs. We further detected the frequency of VISTA expression on PD-1+/Tim-3+/TIGIT+ T cell subsets (**Figures 1B, C**). VISTA+PD-1+ CD3+ (median: 2.56 vs 0.75, *P* < 0.001), CD4+ (median: 3.58 vs 1.10, *P* < 0.001), and CD8+ (median: 1.76 vs 0.48, *P* < 0.001) T cells were increased in MM compared with that in HIs. Similarly, a higher frequency of VISTA+Tim-3+ CD3+/CD4+/CD8+ T cells was found (CD3+ T cells: median: 0.33 vs 0.08, *P* < 0.001; CD4+ T cells: median: 0.24 vs 0.08, *P* < 0.001; CD8+ T cells: median: 0.35 vs 0.07, P < 0.001). In addition, similar increased trends were found for double-positive VISTA+TIGIT+ CD3+ (median: 5.05 vs 1.29, *P* < 0.001), CD4+ (median: 4.66 vs 1.53, *P* < 0.001), and CD8+ T cells (median: 6.19 vs 1.41, *P* < 0.001). Unlike other T cells, Tregs play a role in negative immune regulation; therefore, we characterized the distribution and phenotype of VISTA on Tregs (**Figures 2A, B**). Interestingly, a higher frequency of VISTA and its co-expression with PD-1, Tim-3, and TIGIT was observed in PB in MM compared with HIs (VISTA+ T cells: median: 8.68 vs 3.78, *P* < 0.001; VISTA+PD-1+ T cells: median: 1.67 vs 0.46, *P* < 0.001; VISTA+Tim-3+ T cells: median: 0.41 vs 0.27, *P* = 0.008; VISTA+TIGIT+ T cells: median: 5.97 vs 2.19, *P* < 0.001) (**Figure 2C**).

In addition, we analyzed correlations between the percentages of VISTA and PD-1, Tim-3, and TIGIT on CD3+/CD4+/CD8+/Treg cells in PB from MM patients. The results demonstrated that VISTA is positively correlated with PD-1 expression on CD3+ T cells (rs = 0.416, *P* = 0.012), CD4+ cells (rs = 0.447, *P* = 0.006), and Tregs (rs = 0.332, *P* = 0.048) (**Figure 1D**). Similarly, there was positive correlation between the expression of VISTA and TIGIT on CD4+ T cells (rs = 0.597, *P* < 0.001) and Tregs (rs = 0.545, *P* = 0.001) (**Figures 1D**, **2D**).

### Increased VISTA+CD8+ T cells in BM from patients with MM

**Figure 3:**
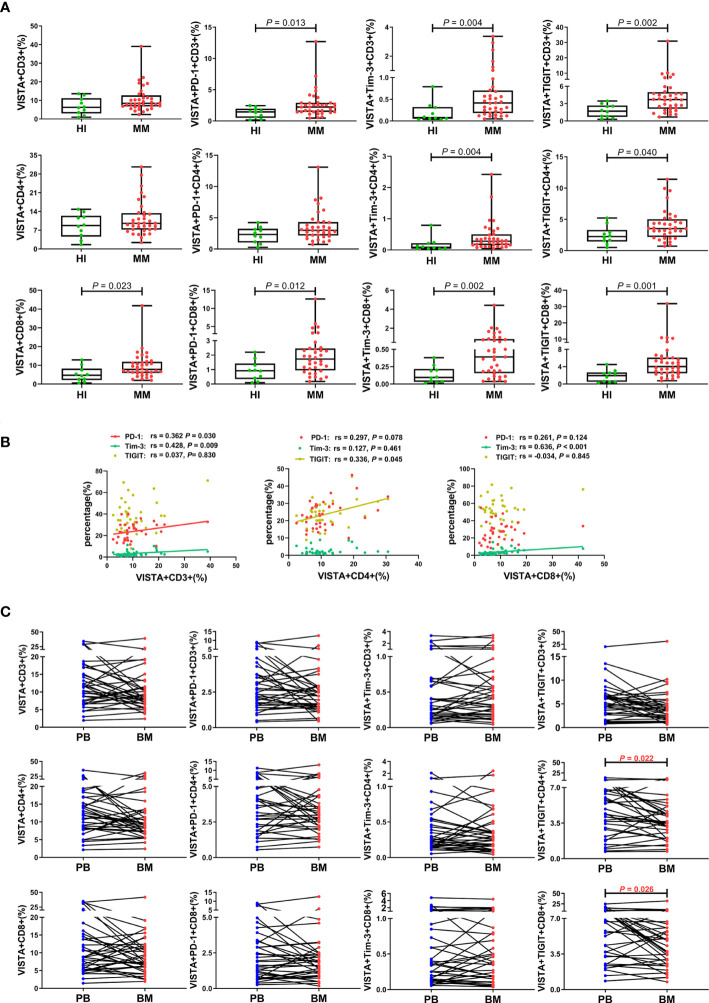
Distribution and frequency of VISTA expression and co-expression with PD-1, Tim-3, and TIGIT on T cell subsets in BM from MM patients. **(A)** Comparison of the frequency of VISTA expression and co-expression with PD-1, Tim-3, and TIGIT on the CD3+, CD4+, and CD8+ T cell subsets in patients with MM and healthy individuals. **(B)** Correlation between the percentages of VISTA+ and PD-1+, Tim-3+, and TIGIT+ T cells on T cell subsets from MM patients. **(C)** Comparison of the percentage of VISTA+, VISTA+PD-1+, VISTA+Tim-3+, and VISTA+TIGIT+ T cells on T cell subsets between PB and BM from 36 patients with MM.

To further compare the different VISTA alterations in PB and BM, we also examined VISTA expression and co-expression with PD-1, Tim-3, and TIGIT on different T cell subsets in BM from patients with MM at the same time (**Figure 3A**). Higher VISTA+CD8+ (median: 7.80 vs 4.69 P = 0.023), VISTA+PD-1+CD8+ (median: 1.73 vs 0.92, *P* = 0.012), VISTA+Tim-3+CD8+ (median: 0.39 vs 0.09, *P* = 0.002), and VISTA+TIGIT+CD8+ (median: 4.04 vs 1.96, *P* = 0.001) T cells were found in MM in comparison with HIs. Interestingly, on CD4+ T cells, VISTA had no significant increase compared to HIs, while the VISTA+Tim-3+ and VISTA+TIGIT double-positive phenotype in CD4+ T cells was statistically increased compared with HIs (median: 0.28 vs 0.09, 3.53 vs 2.25, *P* = 0.004, *P* = 0.040). For VISTA+PD-1+CD4+ T cells, there appeared to be increased expression in the BM for MM patients, but there was no statistically significant difference between groups. With regards to Tregs, there was no significant difference in VISTA+ T cells compared with HIs in BM and no trends towards VISTA co-expression with PD-1, Tim-3, or TIGIT between PB and BM (**Figure 2C**). Furthermore, VISTA expression was found to be positively correlated with PD-1 expression on CD3+ T cells (rs = 0.362, *P* = 0.030), and there was a positive correlation with Tim-3 on CD3+ T cells (rs = 0.428, *P* = 0.009) and CD8+ T cells (rs = 0.636, *P* < 0.001) as well as a positive correlation with TIGIT on CD4+ T cells (rs = 0.336, *P* = 0.045) and Tregs (rs = 0.386 P = 0.020) (**Figures 2D**, **3B**) in BM from MM patients.

We also comparatively analyzed the distributions of VISTA and its co-expression with PD-1, Tim-3, and TIGIT on T cells from 36 pairs of PB and BM samples from patients with MM (**Figure 3C**). Surprisingly, the results revealed a trend toward a significant increased percentage of VISTA expression and co-expression with the IC proteins in PB compared with BM, and there was statistical significance for VISTA+TIGIT+ double-positive cells in the CD4+ (median: 4.66 vs 3.53, *P* = 0.022) and CD8+ (median: 6.19 vs 4.04, *P* = 0.026) T cell subsets. Interestingly, there was a discernable increased percentage of single-positive PD-1+, Tim-3+, and TIGIT+ CD3+ T cells in BM compared with PB (*P* < 0.001; *P* = 0.007; *P* = 0.007), which was consistent with our previous studies (**Figure S1**).

### Higher VISTA expression in T cell subsets is closely correlated with clinical indicators

**Figure 4:**
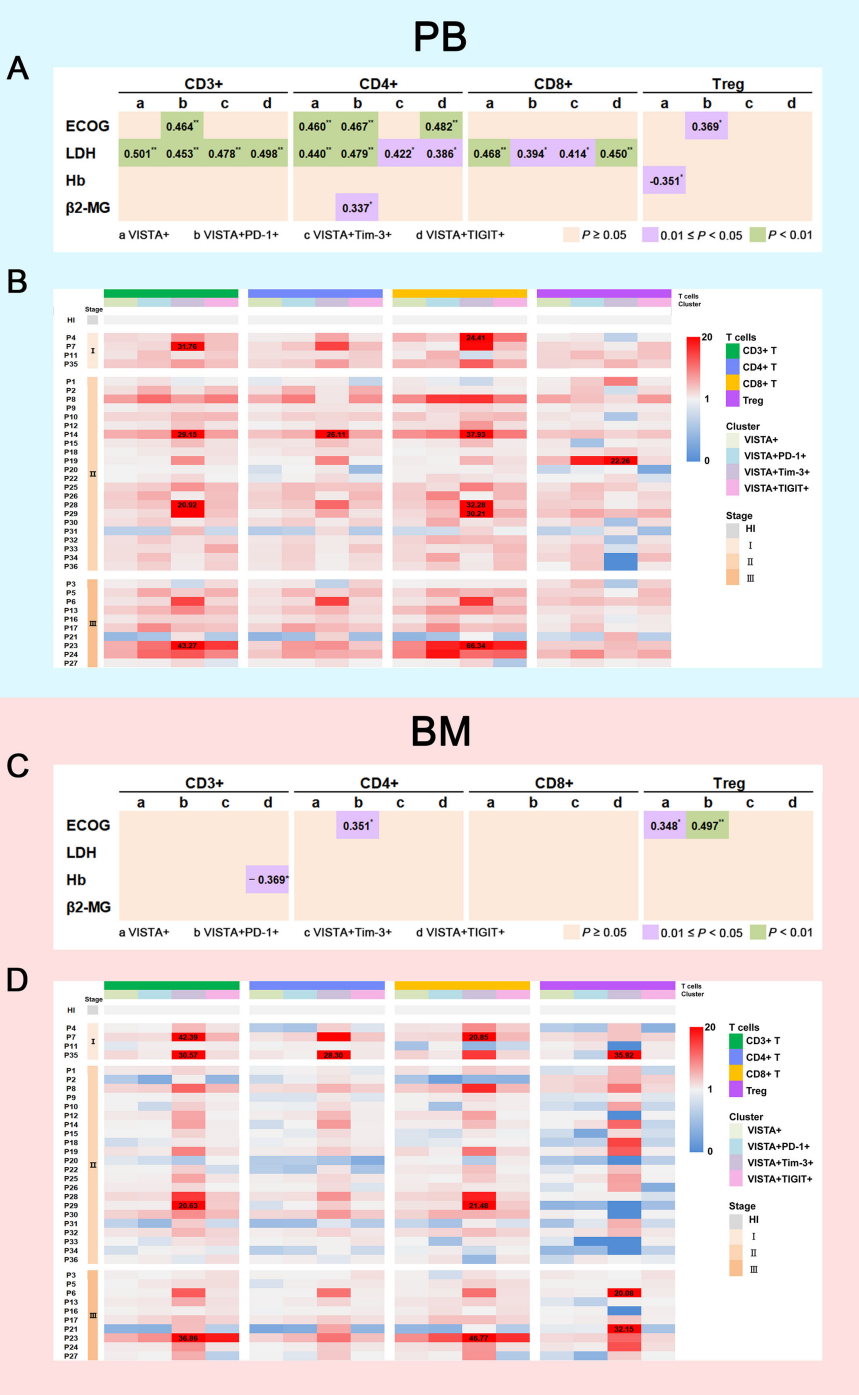
Correlation between VISTA expression on T cell subsets and clinical indicators. **(A)** Correlation between the percentages of VISTA+, VISTA+PD-1+, VISTA+Tim-3+, and VISTA+TIGIT+ T cells on T cell subsets in PB and ECOG scores, LDH, Hb, and β2-MG levels (*P < 0.05; **P < 0.01). **(B)** Heatmap representing the frequency of VISTA+, VISTA+PD-1+, VISTA+Tim-3+, and VISTA+TIGIT+ T cells on T cell subsets in PB from MM patients of different R-ISS stages compared with HIs. **(C)** Correlation between the percentages of VISTA+, VISTA+PD-1+, VISTA+Tim-3+, and VISTA+TIGIT+ T cells on T cell subsets in BM and ECOG score, LDH, Hb, and β2-MG levels. **(D)** Heatmap representing the frequency of VISTA+, VISTA+PD-1+, VISTA+Tim-3+, and VISTA+TIGIT+ T cells on T cell subsets in BM from MM patients of different R-ISS stages compared with HIs.

To explore the relationship between VISTA expression and clinical prognosis, we analyzed VISTA expression, disease stage, and clinical indicators. First, we collected clinical indicator data including Eastern Cooperative Oncology Group (ECOG) scores, lactate dehydrogenase (LDH) levels, hemoglobin (Hb) levels, and beta2-microglobulin (β2-MG) levels and analyzed their correlation with VISTA expression. We used three different color blocks to represent three P value ranges for correlation as shown in **Figures 4A, C**. According to ECOG score, there were significant positive correlations in VISTA+CD4+ (rs = 0.460, *P* = 0.005), VISTA+PD-1+CD3+/CD4+/Treg (CD3+: rs = 0.464, *P* = 0.004; CD4+: rs = 0.467, *P* = 0.004; Treg: rs = 0.369, *P* = 0.027), and VISTA+TIGIT+CD4+ T cells (rs = 0.482, *P* = 0.003) in PB in MM. For BM, similar relationships were found for VISTA+ Treg (rs = 0.348, *P* = 0.038) and VISTA+PD-1+CD4+/Treg (CD4+: rs = 0.351, *P* = 0.036; Treg: rs = 0.497, *P* = 0.002) T cells. Second, VISTA expression was found to be positively correlated with the β2-MG protein level in VISTA+PD-1+CD4+ T cells in PB (rs = 0.337, *P* = 0.045), but there was no statistically significant difference in BM. With regards to Hb, there was a notable negative correlation in VISTA+ Tregs in PB (rs = -0.351, *P* = 0.036) and a negative correlation in VISTA+TIGIT+CD3+ T cells in BM (rs = -0.369, *P* = 0.027). Lastly, we detected the relationship between VISTA expression and LDH in PB. The results demonstrated a positive correlation in VISTA+CD3+/CD4+/CD8+ (CD3+: rs = 0.501, *P* = 0.002; CD4+: rs = 0.440, *P* = 0.007; CD8+: rs = 0.468, *P* = 0.004), VISTA+PD-1+CD3+/CD4+/CD8+ (CD3+: rs = 0.453, *P* = 0.006; CD4+: rs = 0.479, *P* = 0.003; CD8+: rs = 0.394, *P* = 0.018), VISTA+Tim-3+CD3+/CD4+/CD8+ (CD3+: rs = 0.478, *P* = 0.003; CD4+: rs = 0.422, *P* = 0.010; CD8+: rs = 0.414, *P* = 0.012), and VISTA+TIGIT+CD3+/CD4+/CD8+ (CD3+: rs = 0.498, *P* = 0.002; CD4+: rs = 0.386, *P* = 0.020; CD8+: rs = 0.450, *P* = 0.006) T cells. We further followed up on the patients and divided them into stage I, II, and III groups according to the Revised International Staging System (R-ISS) staging criteria for multiple myeloma. The results did not demonstrate a significant trend among disease stage towards VISTA expression in PB (**Figure 4B**). However, a higher percentage of VISTA+TIGIT+ Treg cells was found in the stage III group in comparison with the stage II group in BM (median: 7.69 vs 4.01, *P* = 0.025) (**Figure 4D**).

## Discussion

MM is a common type of hematological malignancy with overall poor prognosis, and T cell immunodeficiency plays an important role in disease progression. Increased expression of IC proteins, such as PD-1, Tim-3, Lymphocyte activation gene 3 (LAG-3), and CTLA-4, resulting in T cell exhaustion is the major reason for T cell immunodeficiency in MM. There is increasing evidence that multiple immune inhibitory receptors participate in T cell exhaustion leading to functional immunosuppression (8). Thus, it is thought that upregulation of IC proteins may be a critical reason for tumor cell immune escape (8, 37–39). However, clinical trials have indicated that the effects of PD-1 blockade are limited as an immunotherapy for MM (40, 41). Further exploration of the phenotypic characteristics of IC proteins may help facilitate designing precision immunotherapies for MM. Recently, VISTA has been regarded as an active immunotherapeutic target in hematologic malignancies; however, little is known about its expression on T cells in MM. In this study, we first analyzed the distribution of VISTA on T cell subsets and found increased VISTA expression in PB from patients with MM, which is consistent with findings in solid tumors (25). In addition, we evaluated VISTA expression concurrent with phenotypically exhausted T cell subsets and confirmed an increase in double-positive VISTA+PD-1+, VISTA+Tim-3+, and VISTA+TIGIT+ CD3+/CD4/CD8+ T cells in PB, indicating that VISTA is closely associated with the induction and development of exhausted T cells. It is known that T cell dysfunction is related to the cancer immune microenvironment (19). In previous studies, researchers have observed a greater impact on T cell suppression in BM compared with that in PB in leukemia and MM patients, which was evident from the higher percentage of PD-1+/Tim-3+ T cells in the BM (4, 42, 43). Interestingly, these results unlike the findings of higher PD-1+/Tim-3+ T cells and TOX+ T cells in BM compared with that in PB in MM patients (10, 42, 43). In this study, we found a higher trend of VISTA+ T cells and VISTA+PD-1+/Tim-3+/TIGIT+ T cells present in PB compared to BM and an even higher percentage of PD-1+, Tim-3+, and TIGIT+ T cells was confirmed in BM compared with PB in MM patients, and the latter is consistent with previous findings (8, 39). This varying distribution of VISTA in PB and BM suggests that VISTA-associated T cell exhaustion in MM tends to be more pronounced in peripheral T cells. The underlying cause of VISTA upregulation needs further investigation.

Tregs, a critical subset of CD4+ T cells that are characterized by the CD4+CD25+FoxP3+ phenotype, play the role of negative immune regulation in solid tumors and hematological malignancies (44). At present, published data regarding Treg number and function are controversial, which is most likely explained by the heterogeneity of experimental approaches and the research objects used (45, 46). It has been reported that Tregs accumulate in MM, and this is closely related to high mortality and reduced survival time, which is consistent with our findings (45, 47, 48). In addition, we previously reported higher expression of immune inhibitory proteins including PD-1, Tim-3, and TOX in Tregs in hematologic malignancies that negatively impact T cell immunosuppressive functions (4, 36, 42). Anna et al. found that patients with MM with higher frequencies of Tregs had inferior survival and a distinct Treg immune checkpoint profile (i.e., increased PD-1, LAG-3) (49). Similarly, we concluded that increased expression of VISTA on Tregs in MM may prompt the activation of Tregs rather than exhaustion. In this study, the up-regulated expression of VISTA concurrent with PD-1, Tim-3, and TIGIT was easily observed in PB compared with BM, suggesting that there is greater Treg activation and T cell immunosuppression.

Up-regulated IC protein expression is closely associated with adverse clinical outcome in cancer and hematological malignancies (23, 50, 51). In this study, we also made the observation that VISTA expression increases with several adverse clinical characteristics, such as advanced ISS stage, lower hemoglobin, and higher levels of β2-MG and LDH, indicating that the development of MM is likely to be related to dynamic changes in the dysfunctional characteristics of VISTA+ T cells. These findings further support a study by Anna et al. who reported that deregulated expression of IC proteins on CD4+ T cells may complicate clinical outcomes and the response to treatment with IC inhibitors in patients with newly diagnosed MM (48). In addition, for patients with adverse clinical characteristics, lower ECOG scores, based on poor quality of life to some extent, demonstrated strong positive correlations with higher VISTA expression in T cell subsets, providing evidence of abnormal VISTA expression towards adverse clinical characteristics. This finding is consistent to the report from Mutaers et al. who has demonstrated that VISTA in the tumor microenvironment is an independent prognostic factor for survival in MM through analysing accessible public database (14). Thus, VISTA expression on T cells plays a critical role in predicting the clinical manifestations and prognoses of patients with MM and emphasizes a superior role of VISTA inhibitors in the development of systemic immune suppression and myeloma progression.

## Conclusions

**Figure 5:**
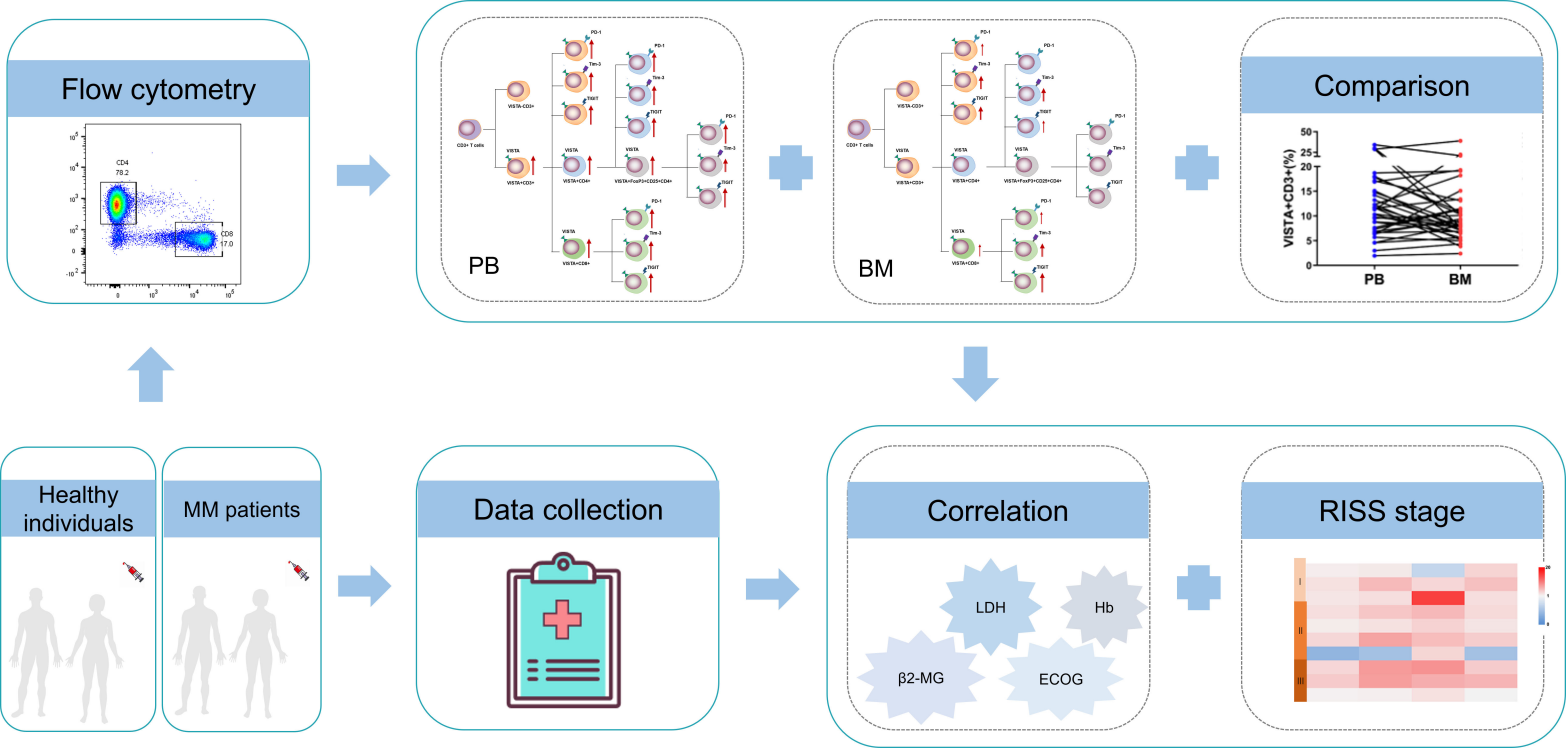
Study schematics and overview of the alterations of VISTA, PD-1, Tim-1, and TIGIT and T cell phenotypes in patients with MM.

In this study, we for the first time made the novel observation that increased VISTA concurrent with PD-1, Tim-3, and TIGIT on T cells is commonly involved in the CD3+, CD4+, Treg, and CD8+ T cell subsets (**Figure 5**). Higher VISTA expression, particularly in PB from patients with MM, may contribute to T cell exhaustion and a damaged anti-tumor response. Furthermore, VISTA was found to be closely associated with disease progression and clinical indicators, suggesting that VISTA may be considered a potential target for reversing T cell exhaustion and improving T cell function in MM.

## Data availability statement

The original contributions presented in the study are included in the article/**Supplementary Material**. Further inquiries can be directed to the corresponding authors.

## Ethics statement

The studies involving human participants were reviewed and approved by the Ethics Committee of School of Medicine of Jinan University and Guangdong Provincial People’s Hospital. The patients/participants provided their written informed consent to participate in this study.

## Author contributions

LZ, YL, and SC contributed to the concept development and study design. SH, YZ, PL, and ZL performed the experiments. JW, XZ, and JT collected the clinical data. YZ and SH contributed to data analysis and figure preparation. LZ, YL, SC, and SH drafted the manuscript. All authors read and approved the final manuscript.

## Funding

This study was supported by grants from the National Natural Science Foundation of China (grant numbers 82070152, 81770152, and 81570143), Research Grant of Key Laboratory of Regenerative Medicine, Ministry of Education Jinan University (grant number: ZSYXM202102) and the Training Program of Innovation and Entrepreneurship for Undergraduates (grant number: CX22448).

## Acknowledgments

We want to thank the flow facility of the Analysis and Testing Center in Jinan University as well as the research assistant Mr. Xinqiang Lai, who helped arrange the study. We would also like to thank the volunteers who donated blood for this project.

## Conflict of interest

The authors declare that the research was conducted in the absence of any commercial or financial relationships that could be construed as a potential conflict of interest.

## Publisher’s note

All claims expressed in this article are solely those of the authors and do not necessarily represent those of their affiliated organizations, or those of the publisher, the editors and the reviewers. Any product that may be evaluated in this article, or claim that may be made by its manufacturer, is not guaranteed or endorsed by the publisher.

## Supplementary material

Supplementary Figure 1 Comparison of the percentages of the PD-1+, Tim-3+, and TIGIT+ T cells in T cell subsets between PB and BM from 36 patients with MM.

## Abbreviations

β2-MG, beta-2-microglobulin; BM, bone marrow; CTLA-4, cytotoxic T-lymphocyte antigen-4; EAC, esophageal adenocarcinoma; ECOG Eastern Cooperative Oncology Group; Hb, hemoglobin; HIs, healthy individuals; IC, immune checkpoint; LAG-3, lymphocyte activation gene 3; LDH, lactic dehydrogenase; MAPK, mitogen-activated protein kinase; MDSC, myeloid-derived suppressor cells; MM, multiple myeloma; mTOR, mammalian target of rapamycin; NSCLC, non-small-cell lung cancer; OS, overall survival; PB, peripheral blood; PD-1, programmed cell death receptor-1; PD-L1, programmed death protein ligand 1; RISS, Revised International Staging System; TCR, T-Cell Receptor; TIGIT, T cell immunoglobulin and ITIM domain; Tim-3, T cell immunoglobulin mucin-domain-containing-3; Treg, regulatory T; VISTA, V-type immunoglobulin domain-containing suppressor of T cell activation.
