# Supplementary figures and images for: Different expression patterns of VISTA concurrent with PD-1, Tim-3, and TIGIT on T cell subsets in peripheral blood and bone marrow from patients with multiple myeloma
Source: Front Oncol. 2022 Nov 10;12:1014904. doi: 10.3389/fonc.2022.1014904 (PMC9684650; doi:10.3389/fonc.2022.1014904)

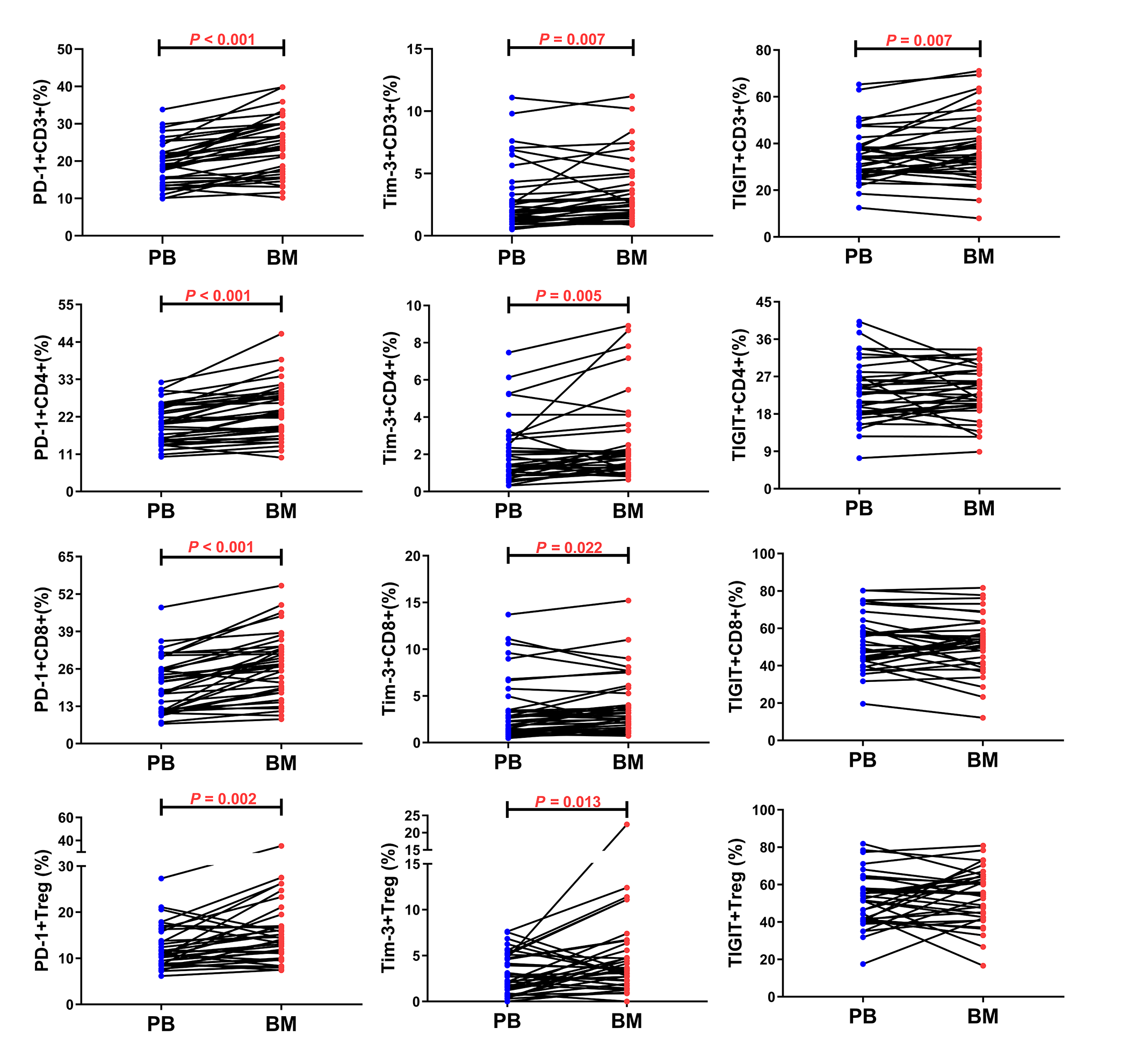

Supplement: Supplementary Figure 1 — Comparison of the percentages of the PD-1+, Tim-3+, and TIGIT+ T cells in T cell subsets between PB and BM from 36 patients with MM. [file Image_1.tif]
