# Supplementary material for: Different expression patterns of VISTA concurrent with PD-1, Tim-3, and TIGIT on T cell subsets in peripheral blood and bone marrow from patients with multiple myeloma
Source: Front Oncol. 2022 Nov 10;12:1014904. doi: 10.3389/fonc.2022.1014904 (PMC9684650; doi:10.3389/fonc.2022.1014904)
Supplement: Supplementary file 2 [file Table_1.docx]

**Supplementary Table 1 Clinical information of MM patients used in the study**

| **Case No.** | **Sex** | **Age** | **MC** | **R-ISS** | **BM proplasmacyte cell (%)** | **ECOG performance status** | **LDH (U/L)** | **Hb**  **（g/L）** | **β2-MG**  **(mg/L)** | **Cytogenetic risk profile**  **assessed by FISH*** | **Therapy** | **Response** |
| --- | --- | --- | --- | --- | --- | --- | --- | --- | --- | --- | --- | --- |
| P1 | F | 58 | IgG | II | 50 | 1 | 133 | 61 | 4.97 | SR | IRD | PR (2021/09/27) |
| P2 | M | 85 | IgG | II | 22 | 3 | 172 | 110 | 4.082 | SR | VD | / |
| P3 | F | 36 | IgG | III | 48.5 | 1 | 82 | 61 | 7.61 | HR | IRD | / |
| P4 | M | 66 | Light chain | I | >10（Pathology） | 1 | 175 | 134 | 1.89 | SR | VRD | VGPR(2022/01/20) |
| P5 | F | 68 | IgG | III | 32 | 1 | 277 | 65 | 15.24 | HR | VCD | / |
| P6 | F | 57 | IgA | III | 53.5 | 1 | 255 | 87 | 14.39 | SR | VRD | CR (2022/05/10) |
| P7 | F | 76 | IgG | I | 11 | 2 | 236 | 117 | 2.79 | SR | RD | / |
| P8 | F | 46 | IgA | II | 40.5 | 1 | 130 | 49 | 4.47 | HR | VRD | PD (2022/06/05) |
| P9 | M | 46 | IgG | II | 22.5 | 1 | 94 | 107 | 2.47 | SR | / | / |
| P10 | M | 56 | IgG | II | 28 | 1 | 156 | 70 | 10.97 | SR | VCD | VGPR (2022/04/26) |
| P11 | M | 62 | IgA | I | 38.5 | 2 | 132 | 112 | 2.7 | SR | IRD | VGPR (2021/07/30) |
| P12 | M | 44 | IgG | II | 39 | 1 | 119 | 99 | 3.48 | SR | VCD | / |
| P13 | F | 62 | IgG | III | 55 | 1 | 433 | 67 | 11.26 | SR | VCD | VGPR (2022/03/26) |
| P14 | F | 36 | Light chain | II | >10（Pathology） | 2 | 344 | 90 | 1.74 | SR | VRD | CR (2022/05/26) |
| P15 | F | 58 | IgG | II | 52 | 1 | 142 | 83 | 3.97 | SR | VRD | CR (2022/05/19) |
| P16 | M | 72 | Light chain | III | 30 | 1 | 142 | 76 | 6.53 | SR | ITD | VGPR (2022/06/24) |
| P17 | F | 62 | IgG | III | 38 | 3 | 161 | 63 | 13.44 | SR | VCD | MR (2021/08/06) |
| P18 | M | 61 | IgG | II | 39.5 | 1 | 64 | 117 | 5.01 | HR | IRD | PR (2022/06/09) |
| P19 | F | 63 | IgG | II | 44.8 | 1 | 105 | 69.4 | 1.934 | HR | PAD,VD | PR (2022/01/18) |
| P20 | M | 68 | IgD | II | 27 | 1 | 125 | 88 | 11.28 | SR | VCD | PR (2022/01/25) |
| P21 | M | 73 | IgA | III | 60 | 1 | 144 | 69 | 3.55 | SR | VCD | / |
| P22 | M | 60 | IgA | II | >10（Pathology） | 1 | 154 | 134 | 1.736 | HR | VTD | CR (2021/12/10) |
| P23 | M | 58 | IgA | III | >10（Pathology） | 1 | 493 | 55 | 118.7 | HR | VCD | sCR (2022/06/14) |
| P24 | M | 64 | IgG | III | 26.5 | 2 | 222 | 83 | 28.5 | SR | VD | PR (2022/02/09) |
| P25 | F | 54 | IgG | II | >10（Pathology） | 1 | 134 | 93 | 4.18 | SR | IRD | VGPR (2022/06/23) |
| P26 | F | 61 | IgA | II | 42 | 1 | 117 | 94 | 4.91 | SR | VRD | sCR (2022/04/14) |
| P27 | M | 41 | IgD | III | 45 | 1 | 122 | 117 | 7.98 | SR | VCD | CR (2022/06/21) |
| P28 | M | 53 | IgG | II | 60 | 2 | 232 | 64 | 31.59 | SR | VCD,VRD | VGPR (2022/06/03) |
| P29 | F | 67 | IgA | II | 19 | 1 | 128 | 69 | 11.62 | HR | VCD | VGPR (2021/11/27) |
| P30 | F | 66 | IgA | II | 10 | 2 | 154 | 102 | 4.58 | HR | VCD | CR (2022/03/18) |
| P31 | F | 57 | IgG | II | 42 | 1 | 176 | 89 | 3.79 | SR | VCD | VGPR (2022/05/18) |
| P32 | M | 68 | IgG | II | >10（Pathology） | 1 | 84 | 110 | 2.2 | HR | IRD | / |
| P33 | M | 72 | IgG | II | 45 | 1 | 222 | 75 | 6.4 | SR | VCD | VGPR (2021/12/20) |
| P34 | M | 73 | IgG | II | 12 | 1 | 247 | 82 | 39.93 | SR | VCD | / |
| P35 | F | 62 | Light chain | I | >10（Pathology） | 1 | 153 | 120 | 2.68 | HR | IRD | / |
| P36 | M | 72 | IgG | II | 39 | 2 | 202 | 91 | 4.49 | SR | ITD | VGPR (2022/06/08) |

Notes: β2-MG: beta2-microglobulin; CR: complete response; ECOG: Eastern Cooperative Oncology Group; FISH: fluorescence in situ hybridization; Hb: hemoglobin; HR: high risk; IRD: ixazomib, lenalidomide, and dexamethasone; ITD: ixazomib, thalidomide, and dexamethasone; LDH: lactate dehydrogenase; MC: monoclonal component; MM: multiple myeloma; MR: minimal response; PAD: bortezomib, doxorubicin, and dexamethasone; PD: progressive disease; PR: partial response; RD: lenalidomide and dexamethasone; R-ISS: Revised International Staging System; SR: standard risk; VCD: venetoclax, carfilzomib and dexamethasone; VD: bortezomib and dexamethasone; VGPR: very good partialre sponse; VRD: bortezomib, lenalidomide, and dexamethasone; VTD: bortezomib, thalidomide, and dexamethasone;

*Cytogenetic risk was assessed by FISH; high risk was defined as the presence of del 17p, p53 mutation, gain 1q, t(4;14), t(14;16) or t(14;20) among patients with available cytogenetic risk data.
