# Supplementary material for: Different expression patterns of VISTA concurrent with PD-1, Tim-3, and TIGIT on T cell subsets in peripheral blood and bone marrow from patients with multiple myeloma
Source: Front Oncol. 2022 Nov 10;12:1014904. doi: 10.3389/fonc.2022.1014904 (PMC9684650; doi:10.3389/fonc.2022.1014904)
Supplement: Supplementary file 3 [file Table_2.docx]

**Supplementary Table 2 The row date for flow cytometry**

| **Sample source** | **Sample number** |
| --- | --- |
| PB in MM | P1-P36 |
| BM in MM | P1-P36 |
| PB in HIs | H1-H36 |
| BM in HIs | H37-H46 |

Note: BM: bone marrow; HIs: healthy individuals; MM: multiple myeloma; PB: peripheral blood.

All row data can be found at: <https://www.jianguoyun.com/p/DeynSGwQ_KHdChiu38oEIAA>
