# Supplementary material for: Different expression patterns of VISTA concurrent with PD-1, Tim-3, and TIGIT on T cell subsets in peripheral blood and bone marrow from patients with multiple myeloma
Source: Front Oncol. 2022 Nov 10;12:1014904. doi: 10.3389/fonc.2022.1014904 (PMC9684650; doi:10.3389/fonc.2022.1014904)
Supplement: Supplementary file 4 [file Table_3.docx]

Supplementary Table 3 The information of antibodies used in this study

| Antibody | Fluorescein | Catalogue Number | Clone | Vendor |
| --- | --- | --- | --- | --- |
| VISTA | PE | 12-1088-42 | B7H5DS8 | eBioscience |
| PD-1- | PE-Cy7 | 561272 | EH12.1 | BD Biosciences |
| Tim-3- | BV421 | 345008 | F38-2E2 | BioLegend |
| TIGIT- | AF647 | 372724 | [A15153G](https://www.biolegend.com/en-us/search-results?Clone=A15153G) | BioLegend |
| CD3- | APC-Cy7 | 557832 | SK7 | BD Biosciences |
| CD4- | BV510 | 344634 | SK3 | BioLegend |
| CD8- | APC-R700 | 565165 | RPA-T8 | BD Biosciences |
| CD25- | BB515 | 564467 | 2A3 | BD Biosciences |
| CD45- | BV605 | 564067 | HI30 | BD Biosciences |
| FoxP3- | BB700 | 566526 | 236A/E7 | BD Biosciences |
| VISTA-ISO | PE | 12-4714-82 | P3.6.2.8.1 | eBioscience |
| PD-1-ISO | PE-Cy7 | 557646 | MOPC-21 | BD Biosciences |
| Tim-3-ISO | BV421 | 400158 | MOPC-21 | BioLegend |
| TIGIT-ISO | AF647 | 400234 | [MOPC-173](https://www.biolegend.com/en-us/search-results?Clone=MOPC-173) | BioLegend |
| CD25-ISO | BB515 | 564416 | X40 | BD Biosciences |
| FoxP3-ISO | BB700 | 566404 | X40 | BD Biosciences |
